# Linear growth beyond 24 months and child neurodevelopment in low- and middle-income countries: a systematic review and meta-analysis

**DOI:** 10.1186/s12887-023-04521-0

**Published:** 2024-02-08

**Authors:** Ravi Prakash Upadhyay, Barsha Gadapani Pathak, Shrish Vijaykumar Raut, Dilesh Kumar, Diksha Singh, Christopher R. Sudfeld, Tor A. Strand, Sunita Taneja, Nita Bhandari

**Affiliations:** 1 Society for Applied Studies, 45 Kalu Sarai, New Delhi, 110016 India; 2 KEM Hospital Research Centre, Pune, India; 3 Christian Medical College, Vellore, India; 4 Harvard T.H Chan School of Public Health, Boston, USA; 5 Innlandet Hospital Trust, Lillehammer, Norway

**Keywords:** Height for age z scores, Length for age z scores, Child development, Cognition, Psychomotor performance, Motor and language performance, Low-and middle-income countries

## Abstract

**Aim:**

To synthesize available evidence on the association between change in linear growth (height for age z score, HAZ) beyond the first two years of life with later child neurodevelopment outcomes in Low- and middle-income countries (LMICs).

**Methods:**

We searched PubMed, Web of Science, and EMBASE for cohort studies on the association between change in HAZ after age two and neurodevelopment outcomes in middle or late childhood. Data extraction was done independently by two reviewers.

**Results:**

A total of 21 studies, that included 64,562 children from 13 LMICs were identified. Each unit increase in change in HAZ above two years is associated with a + 0.01 increase (*N* = 8 studies, 27,393 children) in the cognitive scores at 3.5 to 12 years of age and a + 0.05-standard deviation (SD) increase (95% CI 0.02 to 0.08, *N* = 3 studies, 17,830 children) in the language score at 5 to 15 years of age. No significant association of change in HAZ with motor (standardized mean difference (SMD) 0.04; 95% CI: -0.10, 0.18, *N* = 1 study, 966 children) or socio-emotional scores (SMD 0.00; 95% CI: -0.02, 0.01, *N* = 4 studies, 14,616 participants) was observed.

**Conclusion:**

Changes in HAZ after the first two years of life appear to have a small or no association with child neurodevelopment outcomes in LMICs.

**Supplementary Information:**

The online version contains supplementary material available at 10.1186/s12887-023-04521-0.

## Introduction

Children from low-and middle-income countries (LMICs) settings have been shown to suffer from linear growth faltering, manifested as a high proportion of stunting [1, 2]. Additionally, a substantial number of children from these settings are also documented to not reach their full developmental potential [3, 4]. The largest number of children estimated to not reach their full developmental potential are from sub-Saharan Africa followed by South Asia, which are also regions that experience high levels of stunting [2, 4]. The relationship between linear growth and neurodevelopment in children under two years of life is well documented [5, 6]. Children who are stunted or those with linear growth deficits have been shown to have suboptimal cognitive, psychological language, and motor performance as well as poorer academic performance [7–9]. On similar lines, optimal linear growth in the first two years of life has been shown to be associated with better developmental outcomes. Cognition represents the intellectual abilities of children, such as their intelligent quotient (IQ), executive functions, reasoning skills, and academic abilities like early math or reading skills. Motor development includes both fine and gross motor skills, and it involves milestones like when a child starts to walk. Socioemotional development encompasses aspects like behavior, attachment, emotional expression, and temperament [5]. Language development pertains to a child's ability to communicate thoughts and feelings using words and symbols that are part of their community's native language [10]. A meta-analysis of 68 studies from 29 LMICs showed that each unit increase in HAZ score for children ≤ 2 years was associated with a 0.22-SD increase in cognition at 5 to 11 years [5]. This review focused on cross-sectional studies, exploring the connection between length of age z scores (LAZ) or HAZ scores and neurodevelopment. However, it did not provide summary estimates for studies for examining the relationship between changes in HAZ or height over time and child development [5].

It is believed that in the early formative years of life, both poor linear growth and sub-optimal neurodevelopment share overlapping causes such as inadequate nutrition, high burden of infections and hospitalization, and sub-optimal care at home [11]. In infancy, deficiencies in essential micronutrients like iodine, zinc, iron, copper, vitamin B12, and choline can impede neurocognitive development. Neonatal iodine deficiency is associated with compromised mental abilities, including cretinism in severe cases, and can lead to a 10–15-point reduction in population-level IQ. Insufficient iron intake early in life can result in irreversible behavioral issues [12–14]. The presence of any of these exposures, either alone or in combination may therefore negatively impact both growth and development. With the publication of recent studies that show that recovery from stunting is possible beyond the first 2–3 years of life, there is growing interest in understanding if this improvement leads to enhanced neurodevelopment in children [15–18]. It may mean that if such an association is established, it may lead to more focus on improving the linear growth of children in later years of life. However, it should be noted that neurodevelopment in children is multi-factorial and influenced by key factors such as home environment, nutrition, environmental hygiene, caregiving practices, opportunities for learning, and morbidity prevention. Linear growth is largely a proxy for nutritional and morbidity status and therefore, efforts to improve linear growth would mean more investment in enhancing the nutritional status and reducing the morbidities with a secondary benefit of improving neurodevelopment, school performance, and adult productivity [11, 19].

Based on evidence from recent studies that show the possibility of recovery from early growth failure, we aimed to examine whether growth catch up after 2 years of age was associated with neurodevelopmental outcomes [20–23]. While there are studies exploring the association between change in child HAZ between early and middle/late childhood and neurodevelopment, given that contemporary literature has shown that there is a possibility of catch-up growth after the initial years of life, there has been no systematic effort to synthesize the evidence on this aspect. More so, the findings from individual studies are not coherent. Therefore, we conducted a systematic review and meta-analysis of observational studies that examined the relationship of change in HAZ with neurodevelopmental outcomes. The review is intended to inform whether interventions that promote growth-catch up after the first two years of life may also impact child development outcomes in LMICs.

## Methods

### Data sources and search strategy

This review was registered in PROSPERO (registration number CRD42022352290). We followed the standard PRISMA (Preferred Reporting Items for Systematic Reviews and Meta-Analyses) guidelines for the conduct of this review [24]. The electronic search aimed at identifying studies published from database inception until 31st December 2022. We used three databases i.e., PubMed®, Web of Science, and EMBASE® to perform a literature search. There were no date or language restrictions. The search strategy used for the three databases has been presented in Additional Table 1. The reference lists of the selected articles were searched manually to identify additional relevant articles.

### Study selection and data extraction

We selected cohort/follow-up studies that included children of 2 years and above and showed the relationship between change in HAZ over time and child development. As a result, studies had to have at least two HAZ measurements and should have examined the association between the later–first HAZ measurement with neurodevelopment outcomes. The neurodevelopment outcomes encompass cognition (IQ, executive functioning, and reasoning skills), motor (gross and fine), language, socioemotional-like behavior, temperament, and social competence of a child. There was no exclusion based on the timing between measurements. Although not the primary exposure of interest, we were also interested in documenting whether a change in stunting status was associated with child development outcomes. Therefore, we also included studies that presented data on the change in stunting status after two years of age and its association with neurodevelopmental outcomes. We excluded studies with cross-sectional or case–control designs and from high-income countries, as defined by the World Bank criteria [25]. The primary outcomes of interest were cognition, motor, language, and socio-emotional performance. In addition, we were interested in other aspects of development, including academic achievements, performance in subjects like math and language, school attendance, and other indicators of human capital, as reported in the included studies. All outcomes were reported at the latest follow-up. For the assessment of cognition, language, motor skills, and socio-emotional domains, a variety of neurodevelopmental tools were utilized [26–32]. All such tools used by the authors for assessment will be reported in the summary table.

We used Covidence systematic review software, Veritas Health Innovation, Melbourne, Australia [33]. Two review authors (BGP, SR) independently screened the titles and abstracts to identify the relevant citations, followed by a full-text review. The data was extracted using a modified version of the Cochrane Effective Practice and Organisation of Care Group data collection checklist (Cochrane EPOC Group 2017) [34]. This included study identifiers, study design, participant characteristics, sample size, tools for outcome assessments, and outcome effects. The disagreements or discrepancies between reviewers were resolved by discussions or by referring to a third senior review author (RPU).

### Data analysis and quality assessment

Analysis was performed using Stata 16 software (TX, USA). We reported standardized mean difference (SMD) along with 95% confidence intervals (CIs). As an apriori decision, we decided to include adjusted reported effect sizes from the studies. We defined a set of important variables that preferably should have been adjusted, based on our reading of prior literature. This included variables such as gender, maternal years of schooling, maternal age, maternal depressive symptoms, paternal years of schooling, socio-economic status, residence (urban or rural), child stimulation at home, birth order, and food security. Different studies used different variables for adjustment. However, those that had adjusted for one or more of the variables in our list were eligible to be included in our meta-analysis. The standardized mean difference (SMD) was used as summary statistic as the studies used different psychometric scales for assessing the outcomes of interest. For studies that reported outcomes, especially the IQ/cognitive scores, as mean difference, we calculated SMD using the reported standard deviation (SD) of the overall sample for that particular outcome. We decided to use the random effects model, using the restricted maximum likelihood method (REML), as the included studies differed in their methodology with respect to outcomes considered and tools for measurement, age at outcome assessment and were from different demographic settings. These differences were bound to create substantial heterogeneity (indicated by I^2^ greater than 50%) and therefore, random effects model was considered to be a reliable analytic choice [35]. In the studies included in our review, there was a significant disparity in follow-up duration. Some studies had relatively short follow-up periods of less than 5 years, while others extended beyond 10 years. Presenting subgroup analysis based on periods of follow-up allowed us to examine how changes in linear growth over time (short and long periods) might impact neurodevelopment. We also provided an overall estimate by including studies, irrespective of the duration of follow-up, to complete the entire picture. This approach allowed us to investigate the dynamic relationship between growth catch-up and neurodevelopmental outcomes across different time frames. Understanding the effects of linear growth catch-up over a short and long term follow up on neurodevelopment can have important clinical and policy implications. For example, it might inform healthcare providers and governments to decide for how long programmatic efforts should be made to improve linear growth in children, with an additional benefit of improving neurodevelopment. Additionally, to examine heterogeneity resulting from these variations in follow-up duration, we categorized the follow-up periods into two groups: those less than 5 years and those exceeding 5 years. Publication bias was assessed using Egger’s test and funnel plots [36]. Assessment of the quality of studies was done using the Newcastle–Ottawa Scale [37].

## Results

**Fig. 1:**
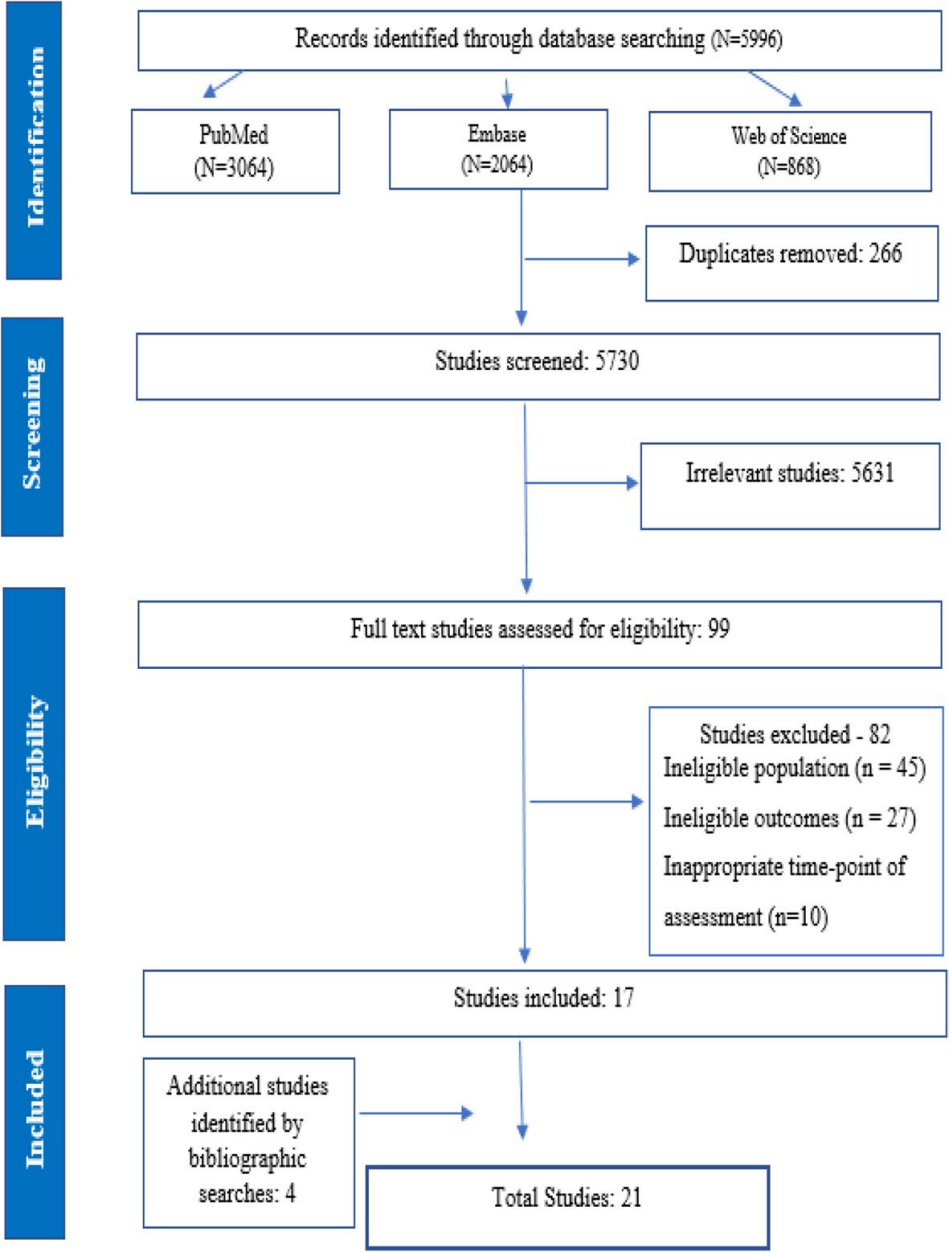
Selection process of studies included in the review

The study flow chart is presented in Fig. 1. Our literature search, as detailed in Supplementary Box 1, initially yielded 5,996 unique citations. After removing 266 duplicates and conducting title and abstract screenings, we proceeded to review the full text of 99 studies. Following this assessment, 82 studies were excluded (see Fig. 1). Subsequently, a bibliographic review of the remaining 17 studies led us to identify an additional 4 relevant studies. As a result, a total of 21 studies were included in this review (Supplementary Table 1). The 21 studies reported on 64,562 children from 13 countries, representing low-income, low-middle-income, and upper-middle-income countries (Table 1). Among the studies that contributed to the quantitative analysis, there were 13 studies that presented data on the cognitive score [16, 18, 38–48], one on motor score [45] and four on language [17, 39, 40, 49] and four on socio-emotional score [39, 44, 45, 48].

**Table 1:** An overview of the included studies

Author (year)/ Country	Outcome assessed/ Assessment tools	Age at baseline and endline assessment of HAZ/LAZ	Sample size	Key variable adjusted in analysis
Upadhyay (2020)/ India [47]	IQ and Executive Functioning/ Wechsler Intelligence Scale for children- Crichton Vocabulary (WISC-CVS) (IQ) and Neuropsychological test battery-II (NEPSY II) for executive functioning	Baseline = 12–36 months Endline = 5–6 years	741	Wealth quintile, number of living children in the family, mother’s years of schooling, father’s years of schooling, father’s occupation
Sokolovic (2014)/ India [18]	Cognitive functioning (IQ)/ Wechsler Intelligence scale of children (WISC/-R/-III)	Baseline = 6 years Endline = 12 years	1,040	Child’s age, and child’s gender
Nguyen (2021)/ Vietnam [44]	Cognitive functioning (IQ) and mental health/ Wechsler Intelligence Scale for Children for Intellectual functioning for IQ and Strengths and Difficulties Questionnaire (SDQ) for mental health	Baseline = 2 years Endline = 7 years	1,392	Maternal (age, parity, and education) and child factors (age, sex, and preschool education), home environment, SES, treatment group, body mass index (BMI) z-scores
Fink (2014)/ India, Peru, Vietnam, Ethiopia [49]	Academic performance; Maths skills; Verbal skills / Peabody Picture Vocabulary Test (PPVT) (Verbal)	Baseline = 8 years Endline = 15 years	3,722	Sex of child, household wealth, caregiver’s age and education
Cheung (2010)/ Philippines [38]	Cognitive functioning (IQ)/ Philippines Non-Verbal Intelligence Test	Baseline = 24 months Endline = 11 years	1,973	Child’s age and gender
Ocansey (2019)/ Ghana [45]	Cognition; motor; language; socio-emotional functioning / NEPSY‐II (cognition and language), NIH toolbox 9-hole pegboard test (motor), SDQ (socioemotional)	Baseline = 18 months Endline = 4–6 years	966	Child age, sex, nulliparity, maternal age, education, height, pre‐pregnancy BMI, marital status, household asset score, household food insecurity index, family care indicator scores, child stimulation (HOME) score, preschool attendance, maternal depressive symptoms and maternal IQ
Poveda (2021) / (Brazil, Guatemala, India, Peru, Philippines and South Africa) [46]	Cognitive functioning (IQ); schooling attainment (Number of years)/ Raven Standard Progressive Matrices for Guatemala, Philippines, South Africa) and WISC for Brazil and Peru);	Baseline = 24 months Endline = 6–9 years	9,503	Maternal measures of height, age of childbirth, and maternal schooling, child birth order, income/wealth quintile
Yang (2011)/ Belarus [39]	IQ and verbal scores; psychosocial behaviours, total difficulties (externalizing, internalizing and pre-social behavior)/ Wechsler Abbreviated Scales of Intelligence (WASI) (IQ and verbal scores), Strengths and Difficulties questionnaire (SDQ) for psychosocial behaviours	Baseline = 12 months Endline = 5 years Cognitive assessment at = 6.5 years	11,899	Sex, gestation (early term/term/post-term), maternal smoking and drinking during pregnancy, duration of breastfeeding, number of older children, parental marital status, parental education and occupation, and parental height, BMI and growth trajectory
Pongcharoen (2012) /Thailand [40]	IQ and verbal scores/ Wechsler Intelligence Scale (IQ/verbal) for Children and the Raven's Colored Progressive Matrices (Pearson) (IQ)	Baseline = 12 months Endline = 9 years	560	Sex, maternal height, mother’s education, socioeconomic status, and location of school
Sunny (2018) /Malawi [50]	School performance (Assessed by a Age-for-grade metric that indicates how many years a child is either ahead of or behind their expected grade level in school, calculated by subtracting their current age from their current grade level, minus 5. This measure offers a comprehensive assessment of a student's educational progress, regardless of the highest grade they have reached)	Baseline = 4 years Endline = 8 years	1,044	Father’s education, mother’s education, household asset index at birth, gender of child and household asset index
Sachdev (2020)/ India [15]	Human capital Metrics (HCM): defined through three metrics: educational status, male occupation, and material possession score	Baseline = 24 months Endline = 5 years	1,184	Socioeconomic status, utilization of health services, maternal and paternal education, paternal occupation, household income, housing condition, and water and sanitation facilities
Adair (2013)/ Brazil, Guatemala, India, Philippines, and South Africa [51]	Completion of secondary school (assessed by the years of the schooling)	Baseline = 24 months Endline = 6–9 years	8,362	Mother’s education and household wealth
Crookston (2010)/Peru [42]	Cognitive functioning (IQ)/ cognitive development assessments (CDAs)	Baseline = 4.5 years Endline = 6 years	1,649	Wealth index and number of siblings
Crookston (2013)/ India, Peru, Vietnam, Ethiopia [52]	Academic performance: mathematics performance; reading comprehension and receptive vocabulary / Early Grade Reading Assessment (reading comprehension),Peabody Picture Vocabulary test (receptive vocabulary)	Baseline = 6 years Endline = 12 years	8,062	Sex of the child, age of the mother, years of schooling of the mother, years of schooling of the father, asset index, urban residence, presence of a community hospital
Casale (2016) /South Africa [16]	Cognitive functioning (IQ)/ Revised Denver Pre-screening Developmental Questionnaire	Baseline = 2 years Endline = 5 years	666	Sex of child, birth weight; SES; birth order, Home environment; Mother/ caregiver inputs in playing/teaching with child
Berkman (2002)/ Peru [41]	Cognition functioning (IQ)/ Weschler’s scale	Baseline = 12 months Endline = 9 years	143	Parental education, child’s grade level (grades 3,4, and 5, combined to reference category), child ever held back in class, and child’s school type
Georgiadis (2017)/ Ethiopia, India, Peru, Vietnam [17]	Mathematics scores and language scores/ Peabody Picture Vocabulary Test (PPVT) (language)	Baseline = 1-year Endline = 12 years	4,723 Ethiopia(1,159), India(1,104),Peru(1,129) and Vietnam(1,331)	Child gender, birth order, child age, caregiver's age at childbirth, ethnicity, and schooling, father's schooling, household monthly per capita expenditure
Sowan (2016)/ Thailand [43]	Cognitive functioning (IQ)/ Nonverbal Intelligence 3rd Edition (TONI-III)	Baseline (HAZ) = 3 years Endline (HAZ) = 5 years Cognitive score at 8.5 years	1,061	Sex, mother’s education, mother’s age, family income, religion, LBW
Gandhi (2011)/ Malawi [53]	Highest school grade completed, number of times repeating a school grade and mathematics scores	Baseline = 1.5- 5 years	325	child’s gender, gestational duration, father’s (occupation and literacy), mother’s literacy and wealth index
Glewwe (2001)/ Philippines [54]	Cognitive functioning (IQ)/ Philippines Non-verbal Intelligence test	Baseline = 2 years Endline = 8 years	1,911	Maternal measures of height, age of childbirth, and maternal schooling, child birth order, income/wealth quintile
Prado (2022)/ Indonesia [48]	Cognitive functioning, and socioemotional scores/ Brief Infant–Toddler Social and Emotional Assessment (Cognitive functioning), Child Behavior Checklist(socioemotional scores)	Baseline = 3.5 years Endline = 9–12 years	359	Child’s gender, socioeconomic status, maternal and paternal years of education, and intervention group

Four studies analyzed data from the Young Lives study [17, 42, 49, 52]. Fink et al. included the older cohort of children aged 8 years and above [49]. Crookston et al. (2010) included children with first/baseline HAZ measured at 6–18 months and endline at 4–6 years and examined the association of change in HAZ with cognitive scores [42]. Crookston et al. (2013), and Georgiadis et al. (2017) included children with baseline HAZ at 12 months and follow-up HAZ at 8 years. These two studies examined the association between HAZ and verbal scores [17, 52]. We expected a substantial, if not complete, overlap of subjects among these two studies and decided to include Georgiadis et. al. (2017) in the quantitative analysis as this was a comparatively recent study [17]. As Crookston et al. (2013) reported on additional outcomes such as academic performance and math scores, we included it for the narrative synthesis of findings [52]. We also reported on few additional outcomes i.e., school overage, academic performance, and human capital metrics which were extracted from some of the included studies [15, 44, 46, 48, 50, 51]. There were 17 good quality studies [9, 15–17, 39, 40, 42–49, 51–53] and remaining four were of fair quality [18, 38, 41, 54] (Supplementary Table 2).

### Cognitive scores

**Fig. 2:**
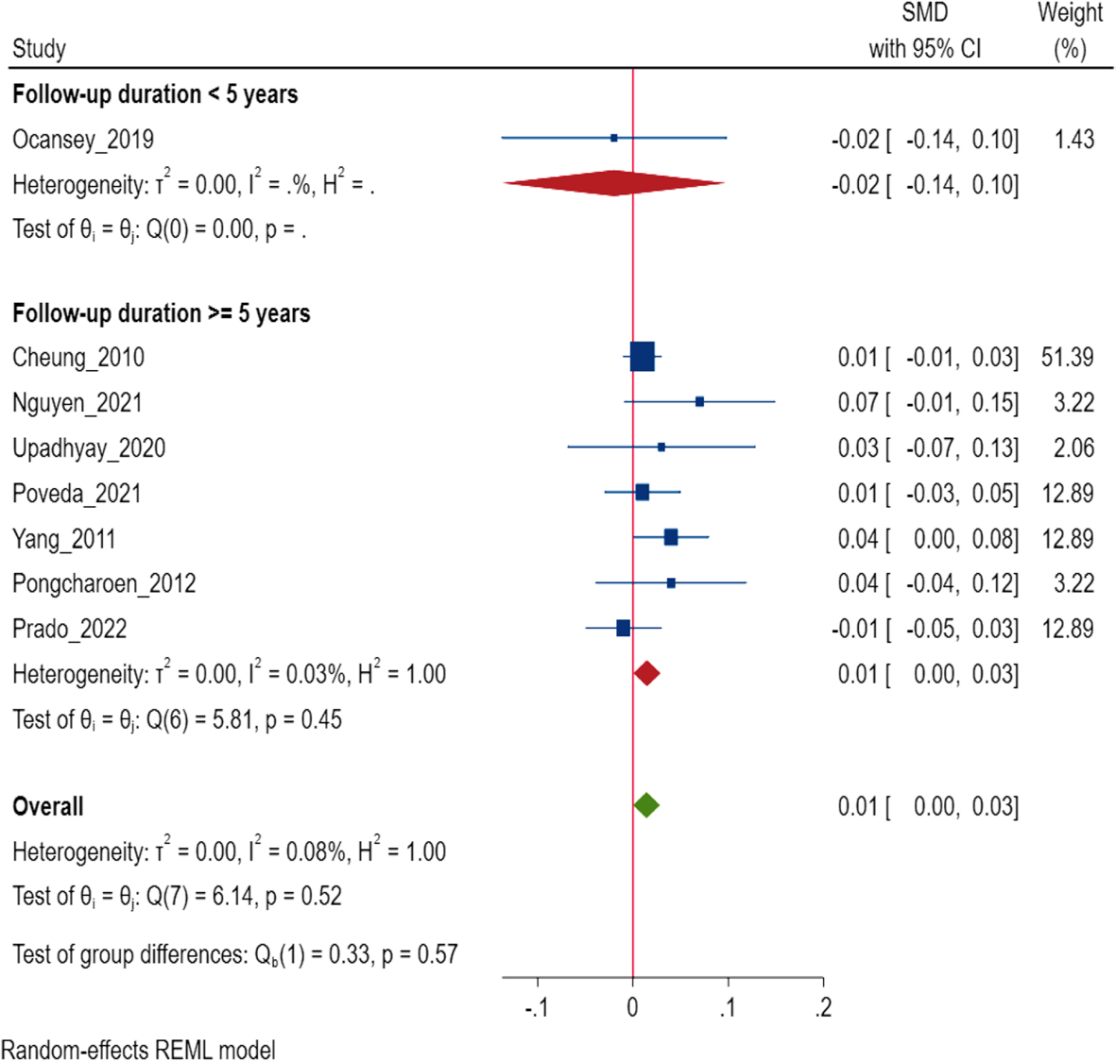
Association of change in height for age z score* (HAZ), with cognitive scores in middle to late childhood. *Some of the studies included in the analysis assessed baseline HAZ/LAZ measurements before the age of 2 years

A total of 8 studies presented the relationship between the change in the HAZ and cognitive scores [38–40, 44–48]. Our analysis noted that each unit increase in change in HAZ was associated with a + 0.01 SD increase in the cognitive score at 3.5 to 12 years of age (95% CI: 0.00, 0.03, I^2^ = 0.08%, *N* = 8 studies, 27,393 participants) (Fig. 2). Egger’s test did not suggest the presence of publication bias (*P* = 0.40) and the funnel plot is presented in Supplementary Fig S1. In the subgroup analysis, we observed one study had a follow-up period of less than 5 years, and was associated with a unit increase in change in HAZ was -0.02 SD (95% CI: -0.14, 0.10, *N* = 1 study, 966 participants) [45] whereas with studies having longer follow up period (≥ 5 years), the pooled effect size was + 0.01 SD (95% CI: 0.00, 0.03, I^2^ = 0.03%, *N* = 7 studies, 26,427 participants) (Fig. 2) [38–40, 44, 46–48].

**Fig. 3:**
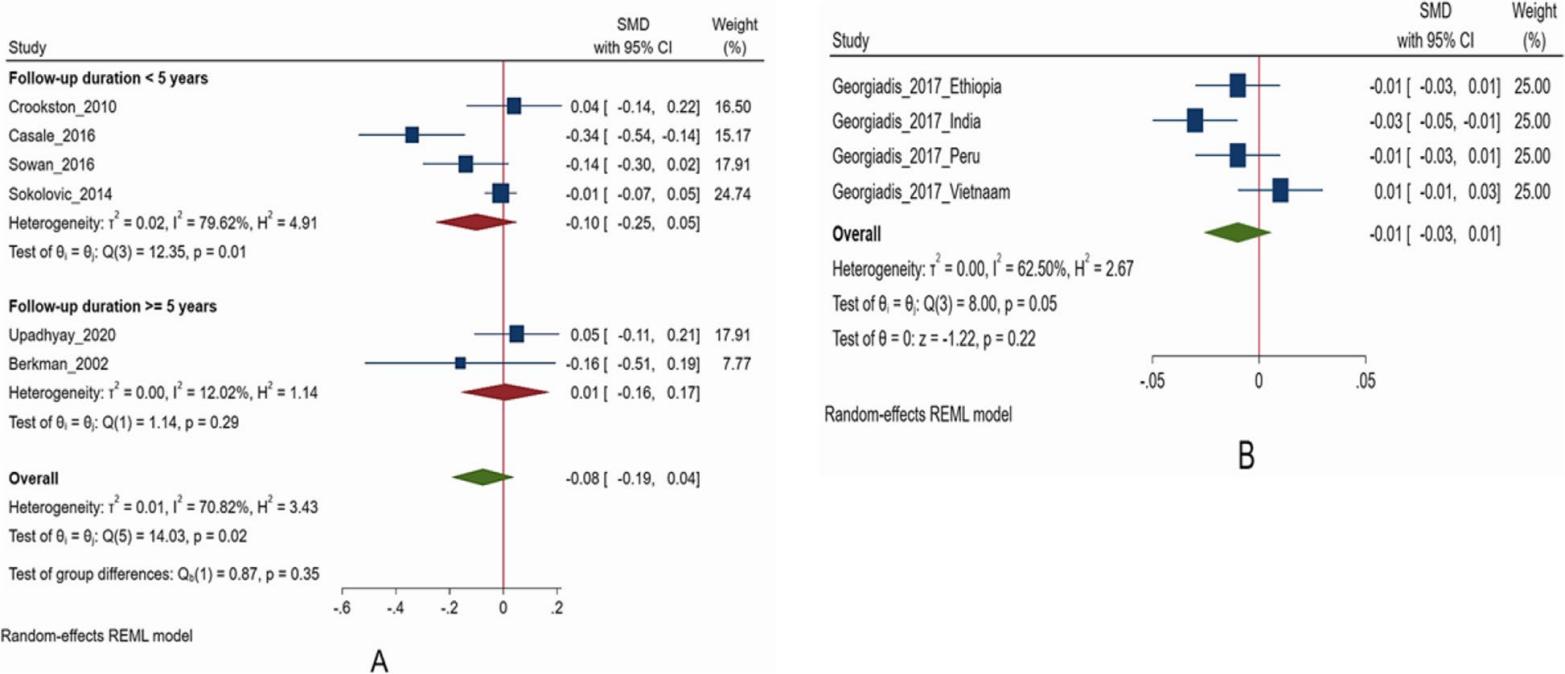
Association of recovery from stunting* with cognitive (**A**) and language scores (**B**), compared to children who were never stunted. *Some of the studies included in the analysis assessed baseline HAZ/LAZ measurements before the age of 2 years

Compared to children who were “never stunted” in the period between early and middle childhood, those who “recovered” from stunting had similar cognitive scores (SMD -0.08; 95% CI: -0.19, 0.04, I^2^ = 70.82%, *N* = 6 studies, 5,300 participants) (Fig. 3) [16, 18, 41–43, 47]. No publication bias was present ( Supplementary Fig S2). On pooling of studies with short (< 5 years) duration of follow up, children who recovered from stunting had similar scores to those never stunted (SMD -0.10; 95% CI: -0.25, 0.05, I^2^ = 79.6%, *N* = 4 studies, 4,416 participants) (Fig. 3) [16, 18, 42, 43]. Similar finding was observed when studies with long duration (≥ 5 years) of follow up were pooled (SMD 0.01; 95% CI: -0.16, 0.17, I^2^ = 12.02%, *N* = 2 studies, 884 participants) (Fig. 3) [41, 47].

### Motor domain scores

Only one study [45] reported on motor domain scores and found that each unit increase in change in HAZ after 18 months of age was not significantly associated with motor score at 4 to 6 years of age (SMD 0.04; 95% CI: -0.10, 0.18, *N* = 1 study, 966 participants).

### Socio-emotional domain scores

**Fig. 4:**
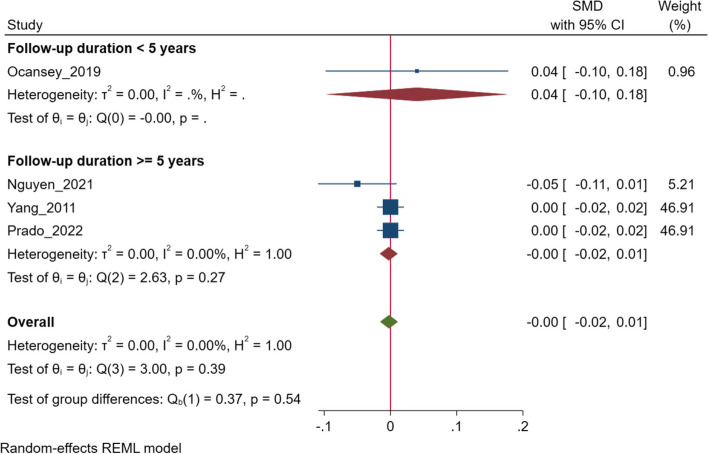
Association of change in height for age z score (HAZ)*, with socio-emotional scores in middle to late childhood. *Some of the studies included in the analysis assessed baseline HAZ/LAZ measurements before the age of 2 years

For the socioemotional domain, estimates were reported in four studies [39, 44, 45, 48]. We noted no association between change in HAZ and socio-emotional scores (SMD 0.00; 95% CI: -0.02, 0.01, I^2^ = 0.00%, *N* = 4 studies, 14,616 participants) in children aged 3.5 to 12 years of age (Fig. 4). Egger’s test did not suggest the presence of publication bias (*P* = 0.61) and the funnel plot is presented is Supplementary Fig. S3. Subgroup analysis based on the duration of follow up also showed findings supporting lack of significant association (Fig. 4).

### Language domain scores

**Fig. 5:**
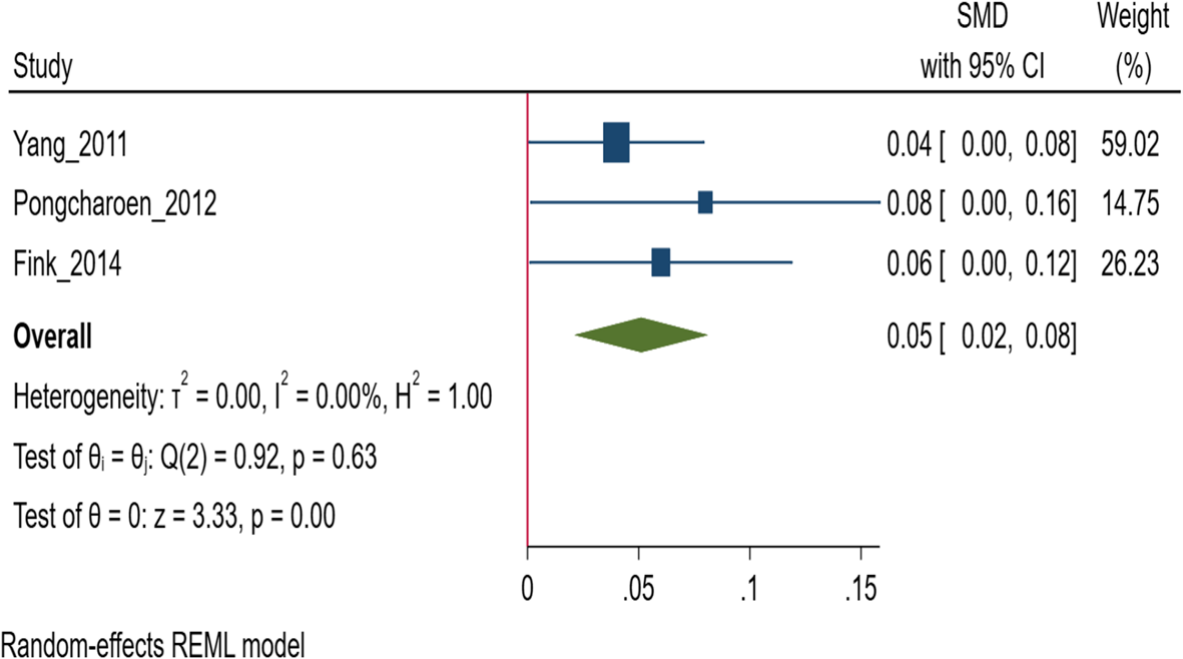
Association of change in height for age z score (HAZ)*, with language scores in middle to late childhood. *Some of the studies included in the analysis assessed baseline HAZ/LAZ measurements before the age of 2 years

Each unit increase in change in HAZ was significantly associated with language scores at ages 5 to 15 years (SMD 0.05; 95% CI: 0.02, 0.08, I^2^ = 0.00%, *N* = 3 studies, 17,830 participants) (Fig. 5) [39, 40, 49]. Egger’s test did not suggest the presence of publication bias (*P* = 0.48) and the funnel plot is presented is Supplementary Fig. S4. Georgiadis (2017) reported the findings from four low- and middle-income countries but did not provide pooled findings [17]. Hence, we have pooled the country-specific data from this study. Compared to children who were “never stunted”, those who “recovered” from stunting had similar scores (SMD -0.01; 95% CI: -0.03, 0.01, I^2^ = 62.5%, *N* = 1 study including 4 countries, 4,723 participants) at 12 years of age (Fig. 3) [17].

### Other measures of development

The association of linear growth improvement beyond the early childhood with academic performance has been explored by some studies and the findings have been mixed. Four of the included studies analysed data from the Young Lives cohort and indicated that improvement in linear growth beyond the first two years may be associated with better academic outcomes [17, 49, 52]. Crookston et al. (2013) evaluated the schooling “overage” that denoted whether a child lagged behind the age-appropriate grade as per country-specific schooling norms. The authors noted that the change in HAZ from 1 to 8 years of age was inversely associated with overage for the grades [52]. Further, an increase in change in HAZ was found to be positively associated with performance in mathematics and reading comprehension [52]. Fink et al. reported that children who were stunted at age 8 years but managed to catch up with their peers by age 15 years, had better mathematics skills and educational achievement than did children who remained stunted [49]. Another Young Lives study found that children who showed persistent recovery from stunting from 1 to 8 years (i.e., those that were not stunted at age 5 and 8 years) performed better than children who remained stunted during this period in mathematical skills [17]. Glewwe et al. examined the impact of change in HAZ from 2 to 8 years on cognitive outcomes in a longitudinal cohort from the Philippines. However, the same cohort was also used in another study by Poveda et al. to assess the association between changes in HAZ and cognitive outcomes. As a result, we have presented the findings from the Glewwe study in Table 1 and decided not to include the study for pooled analysis to prevent data overlap [54].

Sunny et al. found that children who had improved HAZ scores over time (i.e., from 16 months to 8 years) performed better in schools compared to children who were persistently stunted [50]. Adair et al. concluded that faster linear growth between age 2 years to mid-childhood (6 to 9 years) was associated with a reduced risk of not completing secondary school [51]. Poveda et al. evaluated school attainment among children and concluded that linear growth from birth to ∼2 y of age was associated with higher school attainment, but similar associations were not noted for linear growth between 2 years to mid-childhood (6 to 9 years) [46]. Gandhi et al. reported that height gain was positively associated with mathematics test results (*p* = 0.003), reduced grade repetitions (*p* = 0.011), and the highest grade completed (*p* = 0.049) among children who attended school. However, no significant association was found between height gain and the highest grade completed (*p* = 0.194) when children who never attended school were included [53].

Other related aspects of child development were also explored by some studies. For instance, Nguyen et al. concluded that child linear growth, both during and beyond the first 1000 days, was positively associated with mental health during the early school-age years [44]. Sachdev et al. measured human capital metrics consisting of education, male occupation, and material possession [15]. They concluded that height gain from 6 to 24 months was significantly associated with adult education, male occupation, and material possession while height gain after 24 months (till 5 years) was not significantly associated with other components of human metrics except male occupation [15].

Additionally, five studies conducted their baseline assessment of HAZ/LAZ prior to the age of 2 years. Hence, we have undertaken a sensitivity analysis, excluding studies that assessed baseline HAZ measurements before 24 months (Supplementary Fig. S5 to S8). To enhance the transparency of baseline assessment age, we have included a graph that incorporates all studies and their respective baseline assessment ages (Supplementary Fig. S9).

## Discussion

We conducted this systematic review and meta-analysis to primarily understand if the change in linear growth after the first two years of life is associated with improvement in neurodevelopmental outcomes in middle and late childhood. We noted that with each unit increase in change in HAZ, there was a small improvement in cognitive and language score. We observed no association with motor or socio-emotional scores. Additionally, we also found that children who recovered from stunting after the first two years of life had statistically similar cognitive and language scores compared to those who were never stunted. There was also mixed evidence on the relationship of change in HAZ or stunting status with schooling attainment.

Sudfeld et al. in their meta-analysis included cross-sectional studies and found that each unit increase in HAZ among children aged above two years was associated with improvement in scores for cognitive ability (+ 0.09 SD) [5]. Our pooled effect size for association between change in HAZ and cognitive score was comparatively attenuated (+0.01 SD). This could be because we considered the exposure as change in HAZ through inclusion of cohort studies. An interesting observation in our study was that even though the improvement in cognitive and language scores with each unit increase in change in HAZ was small in magnitude, the children who recovered from stunting had similar scores to those who were never stunted. This may mean that those who recovered had their initial HAZ scores nearer to the cut-off for stunting (i.e., -2 SD) and even a small improvement in their HAZ would have had shifted them above the cut-off. Furthermore, this finding could be due to the use of an inaccurate method to measure changes in stunting over time, i.e., the use of a cut-off in HAZ scores to estimate recovery. The inadequacy of this approach has been demonstrated by Leroy and colleagues [19].

Available evidence strongly supports that the first 1000 days (conception through age 24 months) are foundational for brain development [55, 56]. During this period, brain development is rapid with specific neuronal processes occurring over specific time periods. Both adverse and positive experiences during this period critically shape children’s trajectories with respect to health, educational attainment, psychological well-being and economic capacity. Interventions in these early years are critical for brain growth and functioning [57, 58]. In response, many governments and multi-sectoral organizations have begun investing in early child development (ECD) and are promoting programmes addressing children’s development in the early years.

Over the past years, studies have established a fairly strong evidence base to suggest that linear growth in the first two years of life are associated with both concurrent and later childhood neurodevelopment outcomes [6]. The possible explanation for this association could be the shared factors, such as nutrition, repeated infections and morbidities, that influence both these child outcomes [10]. This observed association has been employed to such an extent that some consider linear growth in early childhood to be a proxy for child development. Consequently, public health programs largely invest in improving linear growth with the intent of securing an additional advantage of optimizing cognitive and other aspect of neurodevelopment. There is an added question which largely remains unaddressed systematically i.e., whether there is some potential in the period following the initial two years wherein investments for improving growth could provide dividends with respect to improving child developmental outcomes. Till recent, it was considered that there is limited likelihood of catch-up growth after the first 2–3 years of life as the children continue to remain living in the deprived environments that contributes to continued poor growth [10]. However, contemporary studies have undoubtedly shown that recovery from growth failure can occur [11–14]. Nevertheless, a limitation of this evidence is that few studies have utilized the cut-off HAZ scores for assessing recovery, and this method is an unreliable method [19]. This further demands an exploration of whether this recovery could also improve their cognitive and behavioural functioning as well as academic performance. In focusing on LMICs, our attention is drawn to their disproportionate burden of child stunting—an essential marker of childhood underdevelopment with enduring consequences. The choice of Height-for-Age Z score (HAZ) in this study is deliberate, given its simplicity, cost-effectiveness, and minimal equipment requirements, making it suitable for resource-limited LMICs. HAZ not only reflects early childhood growth but also acts as an early indicator for potential neurodevelopmental delays. Recognizing the challenges in healthcare systems and access to interventions for stunting in these regions, understanding the relationship between HAZ after 2 years and neurodevelopment becomes pivotal. This insight can guide decisions on the timing and cost-effectiveness of interventions, emphasizing the early identification of stunting and the initiation of targeted interventions before the age of 2. By leveraging HAZ, our goal is to promptly identify at-risk children, enabling tailored interventions for enhanced long-term outcomes in these resource-constrained settings.

Prado et al. in their recent meta-analysis showed that nutritional supplementation studies had comparatively higher impacts on linear growth compared to child development outcomes [59]. Further, studies that focused on child stimulation had higher impact on development outcomes compared to growth. Based on these findings, we agree with the authors that the factors that affect linear growth and cognition in later childhood may either not be entirely similar. Consequently, there is a need to specifically target determinants of neurodevelopment rather than attempting to achieve improvement through increase in linear growth.

Through this systematic review and meta-analysis, we also noted that the studies that have conducted long term follow up of children in a systematic and thorough manner are limited. Most studies performed assessments of growth and neurodevelopment at the time of recruitment in the study and at the time of follow-up assessment. One of the reasons could be the limited amount of funding available to carry out such an exercise. The limitation with this nature of assessment and subsequent analysis looking at the relationship between change in linear growth and neurodevelopment outcomes is that the data from the intermediate period is unavailable and information on many of the variables that could influence child neurodevelopment is lacking. Some of these variables include quality of care and stimulation at home, infections and morbidities, transitional changes in anthropometric measures, food security and quality of nutrition. These considerations call for a more supervised and robust follow up wherein data on important variables is captured at frequent intervals.

This is probably one of the first attempts to provide synthesized evidence on the association of “change in linear growth” between early and middle or late childhood and neurodevelopment outcomes. We have reported pooled estimates for this association for a wide range of outcomes i.e., cognitive, language, and socioemotional, as well as recovery from stunting with cognitive and language measures. Moreover, the extensive inclusion of 64,562 children in our study enhances the statistical power of our analysis, while our broad coverage of 13 countries across different income brackets (low, LMICs, and UMIC) enhances the generalizability of our findings. There are some limitations of our analysis that should be considered while interpreting the findings. First, all the included studies were observational in design and therefore, it may not be possible to ascertain a causal link between change in linear growth and neurodevelopmental outcomes. Second, for outcomes other than cognitive performance, the number of available studies were few and therefore, reliable pooled estimates could not be calculated. We noted significant heterogeneity for some of the outcomes. This could be due to differences in the tools measuring neurodevelopment, age at which the children were recruited for baseline assessment, age at follow up assessment, the duration of follow up and variables adjusted for in the statistical analysis. Further, for the cognitive outcome, we included data on a number of related constructs (such as attention, reasoning, IQ) that may have led to some degree of imprecision. An important limitation is the possibility of overlap of children studied in the publications arising out of the Young Lives cohort data [17, 42, 49, 52]. We identified four relevant studies but excluded two studies from the quantitative synthesis because, upon careful examination of the study methods, we found significant overlap of children in this study with another study from the same cohort [17, 52]. However, the possibility that such an overlap still persists among the three remaining studies cannot be overruled. A notable limitation in some studies included in this review is the use of non-validated scales for participant assessment. This calls for caution when interpreting the study findings and drawing conclusions about child neurodevelopment in LMICs. When one uses tools with standards that are not specific to the study population, the direction of association observed may not be impacted; however, the strength of association may not reflect the true estimate. The absence of validated tools and LMIC-specific norms raises concerns that children deemed to have "low" scores may not actually be experiencing developmental delays. These tools might introduce bias by adhering to norms established in high-income countries (HICs), potentially exaggerating the prevalence of delays in LMIC populations. Conversely, the absence of validated tools and LMIC norms may overlook children with genuine developmental challenges, resulting in underdiagnosed and missed opportunities for early intervention. Another possible limitation is that we studied the association of change in HAZ score with selected domains of child development and therefore, the findings may not be applicable to other important aspects of development. Furthermore, the studies included in our analysis provided data on HAZ scores, and there exists a point of contention regarding the concept of catch-up growth within our investigation. This is especially pertinent given that standard deviations for height are not consistent throughout early childhood; they notably increase from birth to 5 years of age. Consequently, when the height for age difference (HAD) (the observed height minus the median height for age according to growth standards) is negative but remains stable as a child ages, the Z-score will actually increase over time. This increase suggests catch-up growth in height, not because the absolute height deficit has diminished, but simply due to the denominator (the standard deviation) increasing [19]. Therefore, it is imperative that future studies investigate the relationship between HAD beyond 24 months of age and the subsequent development of children.

## Conclusion

Based on the findings, we conclude that changes in HAZ during childhood after the first two years of life does not have strong associations with children’s development across domains. Additional follow up studies with robust methodology and periodic data collection on important factors that could influence neurodevelopment is needed to confirm our observations.

### Supplementary Information

**Additional file 1: Supplementary Box 1.** Search Strategy used for identifying relevant studies for the meta-analysis. **Supplementary Table1.** Summary of the included studies. **Supplementary Table 2.** Assessment of quality of the included cohort studies. **Fig S1.** Funnel plot for change in height-for-age z scores over time with cognitive scores. **Fig S2.** Funnel plot for recovery from stunting with cognitive scores. **Fig S3.** Funnel plot for change in height-for-age z scores over time with socioemotional scores. **Fig S4.**  Funnel plot for change in height-for-age z scores over time with verbal scores. **Fig S5.** Sensitivity analysis for association of change in height for age z-score (HAZ), post the first 2 years of age, with cognitive scores in middle to late childhood. **Fig S6.** Sensitivity analysis for association of recovery from stunting with cognitive, post the first 2 years of age, compared to children who were never stunted. **Fig S7.** Sensitivity analysis for association of change in height for age z-score (HAZ), post the first 2 years of age, with socio-emotional scores in middle to late childhood. **Fig S8.** Sensitivity analysis for association of change in height for age z-score (HAZ), post the first 2 years of age, with language scores in middle to late childhood. **Fig S9.** Baseline age of the height/length for age (HAZ/LAZ) assessment among the included studies.

## Data Availability

All the data utilized for the analysis is presented in this manuscript. Any further query could be addressed to the corresponding author.

## Abbreviations

BMI: Body mass index
C.I.: Confidence interval
ECD: Early childhood development
HAZ: Height for age z scores
LAZ: Length for age z scores
HOME: Home observation for measurement of the environment tool
HCM: Human capital metrics
IQ: Intelligence quotient
LBW: Low birth weight
LMIC: Low-Middle-income countries
PRISMA: Preferred reporting items for systematic review and meta-analysis
REML: Restricted maximum likelihood method
SMD: Standardized mean difference
SD: Standard deviation
SES: Socio-economic status
