# Supplementary material for: Linear growth beyond 24 months and child neurodevelopment in low- and middle-income countries: a systematic review and meta-analysis
Source: BMC Pediatr. 2024 Feb 8;24:101. doi: 10.1186/s12887-023-04521-0 (PMC10851505; doi:10.1186/s12887-023-04521-0)
Supplement: Supplementary file 1 — Additional file 1: Supplementary Box 1. Search Strategy used for identifying relevant studies for the meta-analysis. Supplementary Table1. Summary of the included studies. Supplementary Table 2. Assessment of quality of the included cohort studies. Fig S1. Funnel plot for change in height-for-age z scores over time with cognitive scores. Fig S2. Funnel plot for recovery from stunting with cognitive scores. Fig S3. Funnel plot for change in height-for-age z scores over time with socioemotional scores. Fig S4. Funnel plot for change in height-for-age z scores over time with verbal scores. Fig S5. Sensitivity analysis for association of change in height for age z-score (HAZ), post the first 2 years of age, with cognitive scores in middle to late childhood. Fig S6. Sensitivity analysis for association of recovery from stunting with cognitive, post the first 2 years of age, compared to children who were never stunted. Fig S7. Sensitivity analysis for association of change in height for age z-score (HAZ), post the first 2 years of age, with socio-emotional scores in middle to late childhood. Fig S8. Sensitivity analysis for association of change in height for age z-score (HAZ), post the first 2 years of age, with language scores in middle to late childhood. Fig S9. Baseline age of the height/length for age (HAZ/LAZ) assessment among the included studies. [file 12887_2023_4521_MOESM1_ESM.doc]

**SUPPLEMENTARY FILE**

**Title:** **Linear Growth Beyond 24 Months and Child Neurodevelopment in Low- and middle-income Countries: a Systematic Review and Meta-analysis**

Ravi Prakash Upadhyay, MD ^1* †^; Barsha Gadapani Pathak, MD ^1†^; Shrish Vijaykumar Raut, MD ^2^; Dilesh Kumar, MPH ^3^; Diksha Singh, MA ^2^; Christopher R. Sudfeld, ScD ^4^; Tor A Strand, PhD ^5^; Sunita Taneja, PhD ^1^; Nita Bhandari, PhD ^1^

**^†^** Contributed equally towards the first authorship

**Affiliations:** Society for Applied Studies, New Delhi**,** India; ^2^KEM Hospital Research Centre, Pune, India**;** ^3^ Christian Medical College, Vellore, India**;** ^4^Harvard T.H Chan School of Public Health, Boston, United States and ^5^ Innlandet Hospital Trust, Lillehammer, Norway

***Address correspondence to:** Ravi Prakash Upadhyay; Email address: [ravi.upadhyay@sas.org.in](mailto:ravi.upadhyay@sas.org.in); Address: Society for Applied Studies, 45 Kalu Sarai, New Delhi-110016, India; Phone number: 91114604375**Content:**

Supplementary Box 1: Search Strategy used for identifying relevant studies for the meta-analysis

Supplementary Table1: Summary of the included studies

Supplementary Table 2: Assessment of quality of the included cohort studies

Fig S1: Funnel plot for change in height-for-age z scores over time with cognitive scores.

Fig S2: Funnel plot for recovery from stunting with cognitive scores.

Fig S3: Funnel plot for change in height-for-age z scores over time with socioemotional scores.

Fig S4: Funnel plot for change in height-for-age z scores over time with verbal scores.

Fig S5: Sensitivity analysis for association of change in height for age z-score (HAZ), post the first 2 years of age, with cognitive scores in middle to late childhood

Fig S6: Sensitivity analysis for association of recovery from stunting with cognitive, post the first 2 years of age, compared to children who were never stunted

Fig S7: Sensitivity analysis for association of change in height for age z-score (HAZ), post the first 2 years of age, with socio-emotional scores in middle to late childhood

Fig S8: Sensitivity analysis for association of change in height for age z-score (HAZ), post the first 2 years of age, with language scores in middle to late childhood

Fig S9: Baseline age of the height/length for age (HAZ/LAZ) assessment among the included studies

**Supplementary Box 1. Search Strategy used for identifying relevant studies for the meta-analysis**

| **Pubmed**  **(Study population AND Study type) NOT (exclusion) AND (Exposure) AND (Outcome)** |
| --- |
| ("child*"[tiab] OR Child,Preschool [MeSH] ) AND (“Cohort studies”[MeSH] OR “Cross-sectional studies” [MeSH] OR "Randomized Controlled Trial" [Publication Type] OR "Case-Control Studies"[Mesh] OR “Observational studies”[tiab] OR “observational studies” [tw] OR “Preprint”[pt]) NOT (Address[ptyp] OR Autobiography[ptyp] OR Bibliography[ptyp] OR Biography[ptyp] OR pubmed books[filter] OR Case Reports[ptyp] OR Congress[ptyp] OR Consensus Development Conference[ptyp] OR Directory[ptyp] OR Duplicate Publication[ptyp] OR Interview[ptyp] OR Lecture[ptyp] OR Legal Case[ptyp] OR News[ptyp] OR Newspaper Article[ptyp] OR Personal Narrative[ptyp] OR Portrait[ptyp] OR Retracted Publication[ptyp] OR Video-Audio Media[ptyp] OR “Meta-Analysis” [pt] OR “Systematic Review”[pt]) AND (Body height [Mesh] OR Height-for- age [tiab] OR “Height-for-age”[tw] OR “height” [tw] OR Catch-up-growth[tiab] OR “catch-up growth”[tw] OR “Height for age deficit”[tw] OR “HAD” [tw] OR “HAZ” [tw] OR stunt* [tiab] OR “stunt” [tw] OR length-for- age [tiab] OR “length-for-age”[tw] OR “length” [tw] OR “LAZ” [tw]) AND ("Child development" [Mesh] OR "cogniti*"[tw] OR "psychomotor" [tw] OR "sensorimotor"[tw] OR "motor"[tw] OR "intelligence"[tw] OR "IQ"[tw] OR "Intelligence quotient"[tw] OR "language development*" [tw] "language"[tw] or "executive function"[tw] OR "attention" [tw] OR "memory"[tw] OR "learning"[tw] OR "information processing" [tw] OR "literacy" [tw] OR "reading"[tw] OR "math"[tw] OR "school readiness"[tw] OR "pre-academic"[tw] OR "academic"[tw] OR "mental health" [tw] OR "behavior problem" [tw] OR "emotional"[tw] OR "emotion"[tw] OR "socioemotional"[tw] OR "temperament"[tw] OR "self-regulation"[tw] OR "attachment" [tw] OR "self-esteem" [tw] OR "self-efficacy"[tw] OR "social competence" [tw] OR "peer relationship"[tw] OR "prosocial behavior"[tw] OR "internalizing"[tw] OR "externalizing"[tw] OR "hyperactivity"[tw] OR "impulsivity"[tw] OR "aggression"[tw]) |
| **Web of Science** |
| ALL=(“Child*” )or ALL=("Children,Pre-school*") AND TS=("Body height" ) OR ALL=("Height-for- age ") OR ALL=("Height") OR ALL=("catch-up growth") OR TS=("catch-up growth") OR TS=("Height for age deficit") OR ALL=("HAD") OR TS=("HAZ") OR TS=(stunt*) OR ALL=("length-for- age ") OR ALL=("length") OR ALL=("LAZ") OR ALL=("Height for age deficit") AND ALL=(“Child development” )or ALL=(cogniti*) OR ALL=(“psychomotor”) OR ALL=( “sensorimotor”) OR ALL=(“motor”) OR ALL=( “intelligence”) OR ALL=(“IQ”) OR ALL= (“Intelligence quotient”) OR ALL=(executive function*) OR ALL=(“language”) OR ALL=( language development*) OR ALL=(“attention”) OR ALL=(“memory”) OR ALL=(“learning”) OR ALL=(“reading”)OR ALL=(“math”) OR ALL=( mathematic*)OR ALL=("school readiness") OR ALL=( pre-academic*) OR ALL=(academic*) OR ALL=(“mental health”)OR ALL=( behaviour* problem*) OR ALL=(emotion*) OR ALL=(“socioemotional”) OR ALL=(temperament*) OR ALL=(“self-regulation”) OR ALL=( “attachment”) OR ALL=(“self-esteem”) OR ALL=(“externalizing”) OR ALL=(self-efficac*) OR ALL=(“social competence") OR ALL=( peer relationship*) OR ALL=(prosocial behaviour*) OR ALL=(“internalizing”) OR ALL=( hyperactivity*) OR ALL=(impulsivit*) OR ALL=( aggress* ) OR ALL=("literacy") OR ALL=( information processing*) AND ALL=(“Child development” )or ALL=(cogniti*) OR ALL=(“psychomotor”) OR ALL=( “sensorimotor”) OR ALL=(“motor”) OR ALL=( “intelligence”) OR ALL=(“IQ”) OR ALL= (“Intelligence quotient”) OR ALL=(executive function*) OR ALL=(“language”) OR ALL=( language development*) OR ALL=(“attention”) OR ALL=(“memory”) OR ALL=(“learning”) OR ALL=(“reading”)OR ALL=(“math”) OR ALL=( mathematic*)OR ALL=("school readiness") OR ALL=( pre-academic*) OR ALL=(academic*) OR ALL=(“mental health”)OR ALL=( behaviour* problem*) OR ALL=(emotion*) OR ALL=(“socioemotional”) OR ALL=(temperament*) OR ALL=(“self-regulation”) OR ALL=( “attachment”) OR ALL=(“self-esteem”) OR ALL=(“externalizing”) OR ALL=(self-efficac*) OR ALL=(“social competence") OR ALL=( peer relationship*) OR ALL=(prosocial behaviour*) OR ALL=(“internalizing”) OR ALL=( hyperactivity*) OR ALL=(impulsivit*) OR ALL=( aggress* ) OR ALL=("literacy") OR ALL=( information processing*) NOT TS=("Autobiograph*" ) OR TS=("Bibliograph*") OR TS=("Case Reports") OR TS=("Congress") OR TS=("Consensus Development Conference") OR TS=("Directory") OR TS=("Duplicate Publication") OR TS=("Interview*") OR TS=("Lecture" ) OR TS=("Legal Case") OR TS=("News") OR TS=("Newspaper Article*") OR TS=("Personal Narrative")OR TS=("Portrait") OR TS=("Retracted Publication*") OR TS=("Video-Audio Media") OR TS=("Meta-Analysis") OR TS=("Systematic Review") |
| **EMBASE** |
| (exp child/ or exp preschool child/) and (randomized control trial.mp. or exp randomized controlled trial/ or exp cohort analysis/ or cross-sectional analysis.mp. or exp *case control study/ or observational study.mp. or exp observational study/ or pre-print.mp.) and (body height.mp. or exp body height/ or exp height/ or Height-for-age.mp. or exp stunting/ or exp catch up growth/ or length-for-age.mp.) and (child development.mp. or exp child development/ or exp psychomotor disorder/ or exp psychomotor development/ or psychomotor.mp. or exp motor dysfunction/ or exp motor control/ or exp motor development/ or motor.mp. or exp motor learning/ or exp motor coordination/ or exp motor performance/ or exp cognition/ or cogniti.mp. or exp cognitive defect/ or exp intelligence test/ or exp intelligence quotient/ or intelligence.mp. or exp intelligence/ or language development.mp. or exp language development/ or executive funtion.mp. or exp attention/ or attention.mp. or exp attention disturbance/ or exp memory/ or memory.mp. or information processing.mp. or exp information processing/ or literacy.mp. or exp literacy/ or reading.mp. or exp reading/ or exp mathematics/ or school readiness.mp. or pre-academic.mp. or exp academic achievement/ or academic.mp. or mental health.mp. or exp mental health/ or behaviour problem.mp. or exp problem behavior/ or emotional.mp. or exp social behavior/ or exp emotion/ or socio-emotional.mp. or temperament.mp. or exp temperament/ or self-regulation.mp. or exp emotional attachment/ or attachment.mp. or self-esteem.mp. or exp self-esteem/ or self-efficacy.mp. or social competence.mp. or exp social competence/ or peer relationship.mp. or exp social behavior/ or prosocial behavior.mp. or externalizing.mp. or internalizing.mp. or hyperactivity.mp. or exp hyperactivity/ or impulsivity.mp. or exp impulsiveness/ or aggression.mp. or exp aggression/) |

**Supplementary Table 1: Summary of the included studies**

| **Characteristic** | **Number of studies** |
| --- | --- |
| Number of Studies | 21 (64562 children, 13 countries)  India, Malawi, Brazil, Philippines, South Africa, Peru, Vietnam, Ethiopia, Belarus, Ghana, Guatemala, Thailand, Indonesia) |
| Publication dates (From-to) | 1988 - 2022 |
| Study Design |  |
| Cohort | 21 |
| Country Income Group | 13 |
| Lower middle income | 6  (India, Philippines, Indonesia, Vietnam, Ghana, Guatemala) |
| Upper middle income | 5  (Brazil, South Africa, Belarus, Peru, Thailand) |
| Low Income | 2  (Malawi, Ethiopia) |
| Developmental domains assessed |  |
| Cognitive domain  Motor domain  Language  Socio-emotional/Behavioural | 14  1  5  4 |

**Supplementary Table 2.** **Assessment of quality of the included cohort studies**

| **Author/Year** | **Design** | **Representativeness of the Exposed Cohort** | **Selection:**  **non**  **exposed**  **cohort** | **Ascertainment of**  **Exposure** | **Comparability**  **of groups on**  **basis of design**  **or analysis** | **Outcome:**  **Assessment** | **Adequacy**  **of follow-up** | **Total** |
| --- | --- | --- | --- | --- | --- | --- | --- | --- |
| Crookston_2010 | Cohort | * | * | * | * | * | * | 6  (Good quality) |
| Crookston_2013 | Cohort | * | * | * | * | * | * | 6  (Good quality) |
| Cheung_2009 | Cohort | - | * | * | * | * | * | 5  (Fair quality) |
| Casale_2015 | Cohort | * | * | * | * | * | * | 6  (Good quality) |
| Adair_2013 | Cohort | * | * | * | * | * | * | 6  (Good quality) |
| Berkman_2002 | Cohort | - | * | * | * | * | * | 5  (Fair quality) |
| Poveda_2021 | Cohort | * | * | * | * | * | * | 6  (Good quality) |
| Sowan_2016 | Cohort | * | * | * | * | * | * | 6  (Good quality) |
| Sunny_2018 | Cohort | * | * | * | * | * | * | 6  (Good quality) |
| Sachdev_2020 | Cohort | * | * | * | * | * | * | 6  (Good quality) |
| Upadhaya_2019 | Cohort | * | * | * | ** | * | * | 7  (Good quality) |
| Nguyen_2021 | Cohort | * | * | * | ** | * | * | 7  (Good quality) |
| Ocasey_2019 | Cohort | * | * | * | * | * | * | 6  (Good quality) |
| Sokolovic_2014 | Cohort | - | * | * | * | * | * | 5  (Fair quality) |
| Yang_2011 | Cohort | * | * | * | * | * | * | 6  (Good quality) |
| Pongcharen_2012 | Cohort | * | * | * | * | * | * | 6  (Good quality) |
| Fink_2014 | Cohort | * | * | * | * | * | * | 6  (Good quality) |
| Georgiadis_2017 | Cohort | * | * | * | * | * | * | 6  (Good quality) |
| Glewwe_2001 | Cohort | - | * | * | * | * | * | 5  (Fair quality) |
| Gandhi_2011 | Cohort | * | * | * | * | * | * | 6  (Good quality) |
| Prado_2022 | Cohort | * | * | * | ** | * | * | 7  (Good quality) |

*Assessed by New Castle Ottawa scale. It assesses the cohort study in three domains i.e., selection, comparability, and outcome. **Good quality**: 3 or 4 stars in selection domain AND 1 or 2 stars in comparability domain AND 2 or 3 stars in outcome/exposure domain; **Fair quality**: 2 stars in selection domain AND 1 or 2 stars in comparability domain AND 2 or 3 stars in outcome/exposure domain; **Poor quality**: 0 or 1 star in selection domain OR 0 stars in comparability domain OR 0 or 1 stars in outcome/exposure domain

**Funnel plots**

**Fig S1**: Funnel plot for change in height-for-age z scores over time with cognitive scores


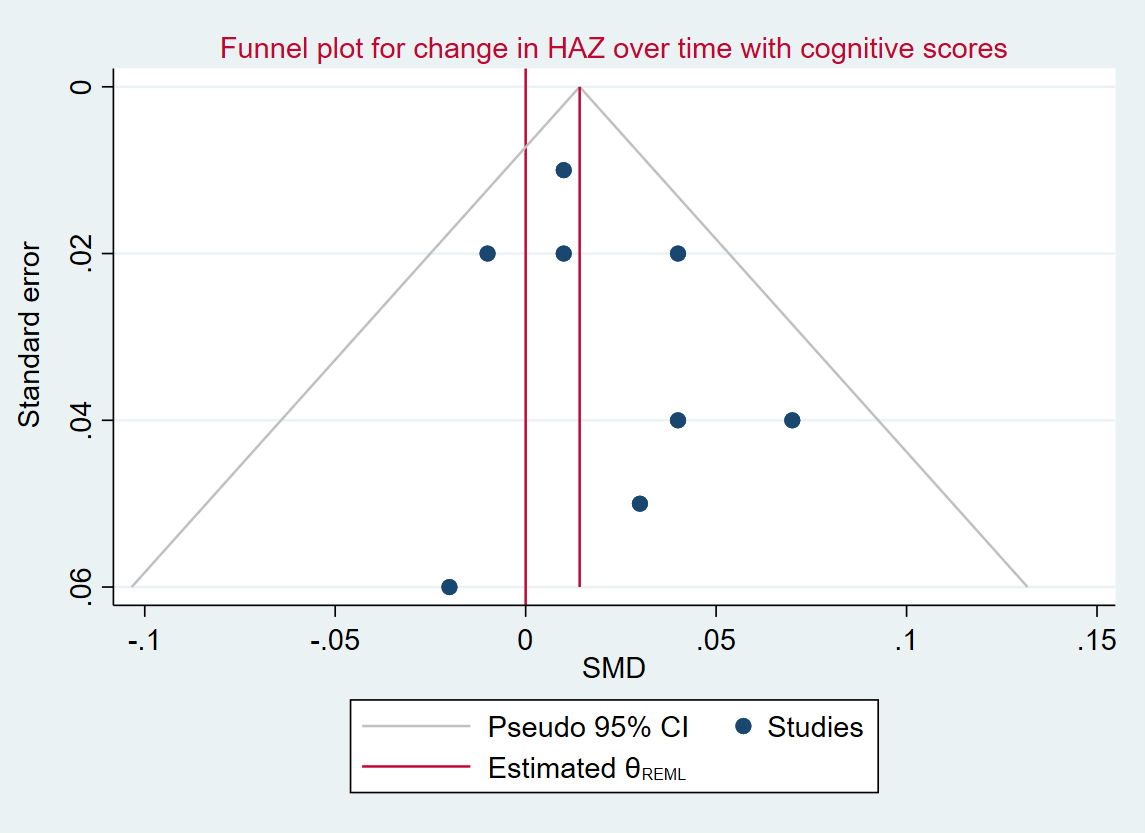


**Fig S2:** Funnel plot for recovery from stunting with cognitive scores


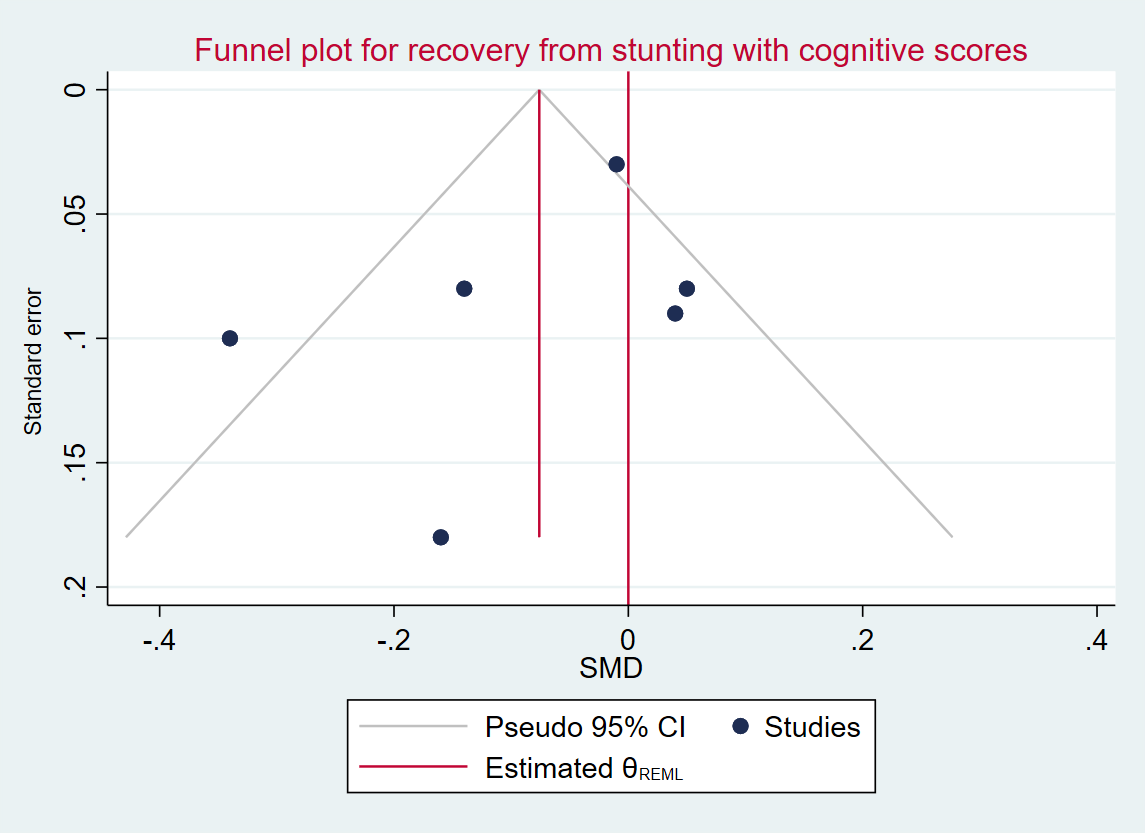


**Fig S3:** Funnel plot for change in height-for-age z scores over time with socioemotional scores


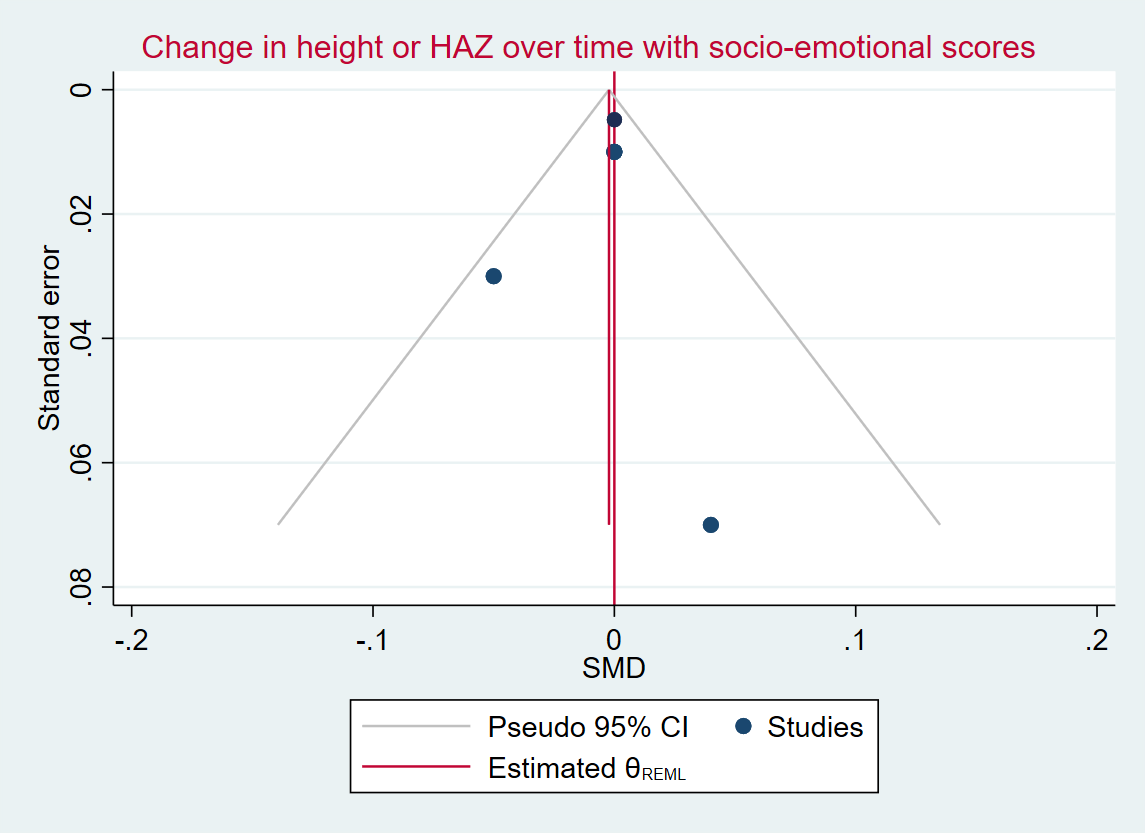


**Fig S4:** Funnel plot for change in height-for-age z scores over time with verbal scores


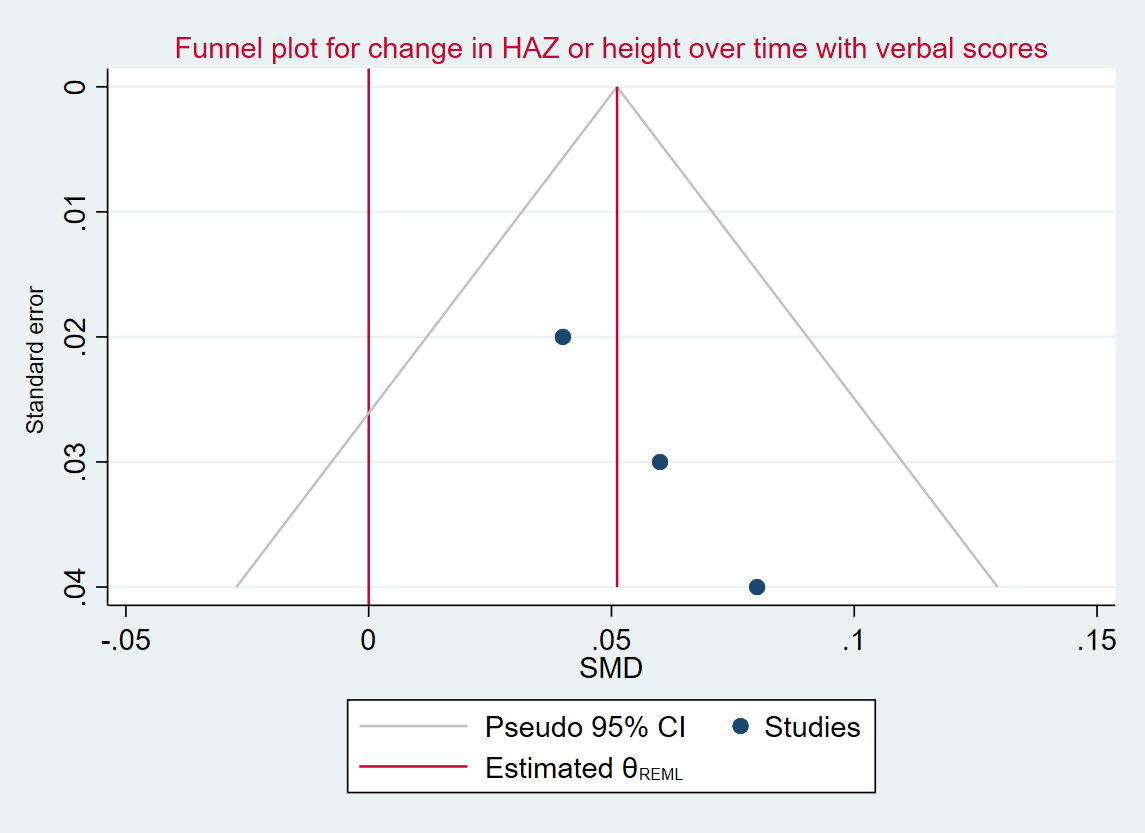


**Sensitivity analysis**

**Fig S5. Association of change in height for age z-score (HAZ), post the first 2 years of age, with cognitive scores in middle to late childhood**

***
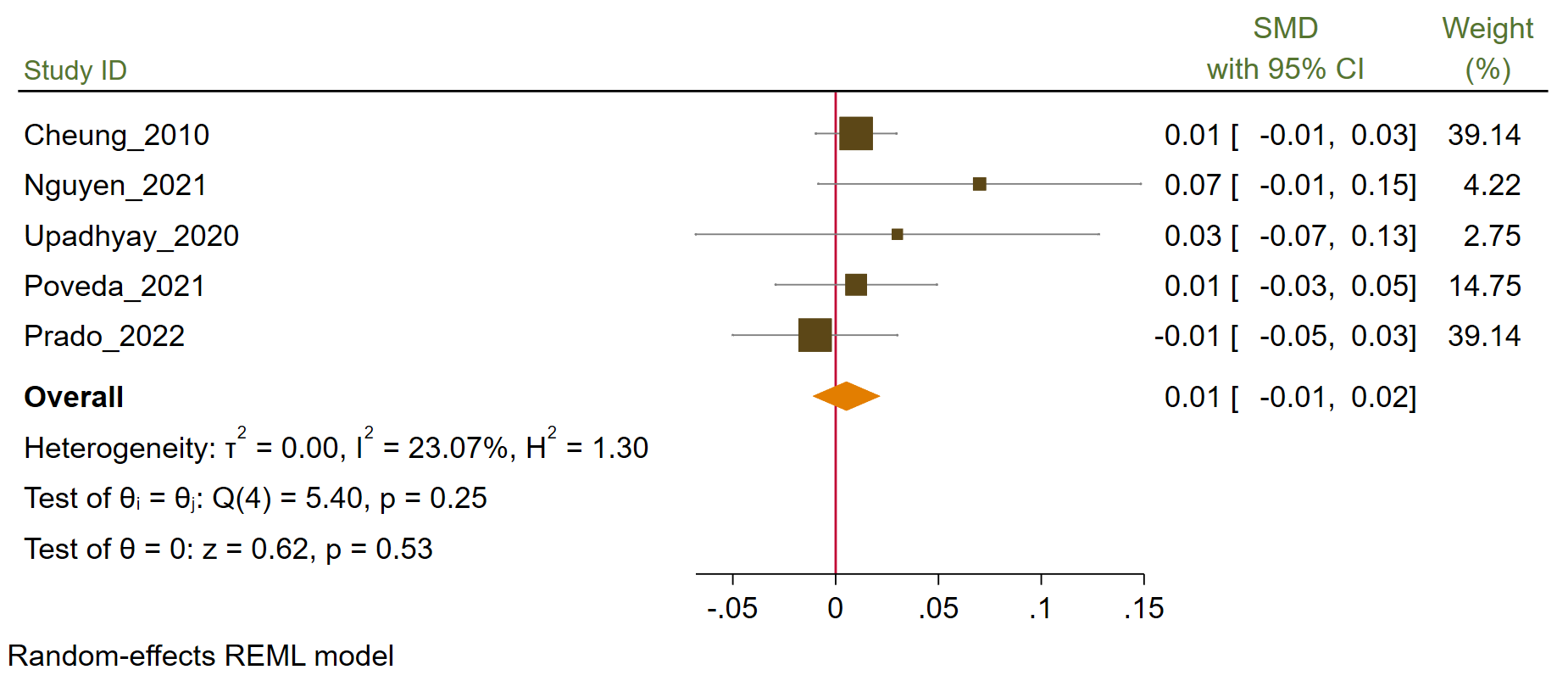
Here all the studies included for pooling have a follow-up period is more than 5 years*.**

**Fig S6. Association of recovery from stunting with cognitive, post the first 2 years of age, compared to children who were never stunted**

**
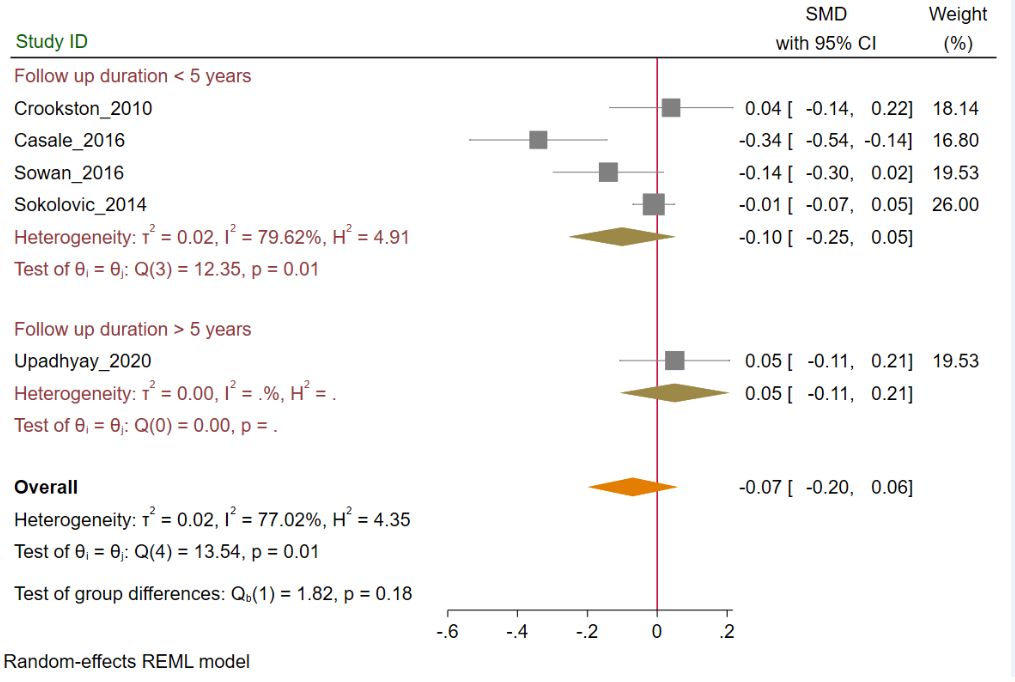
**

**Fig S7. Association of change in height for age z-score (HAZ), post the first 2 years of age, with socio-emotional scores in middle to late childhood**

**
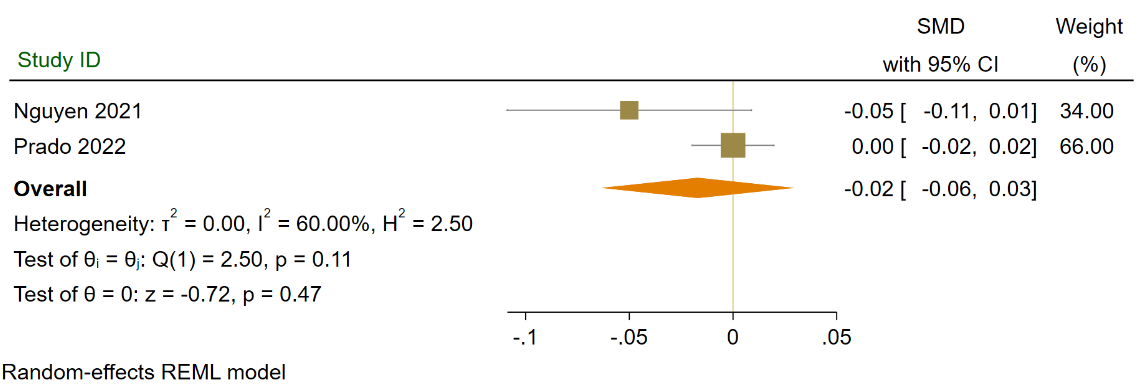
**

**Fig S8. Association of change in height for age z-score (HAZ), post the first 2 years of age, with language scores in middle to late childhood**

**
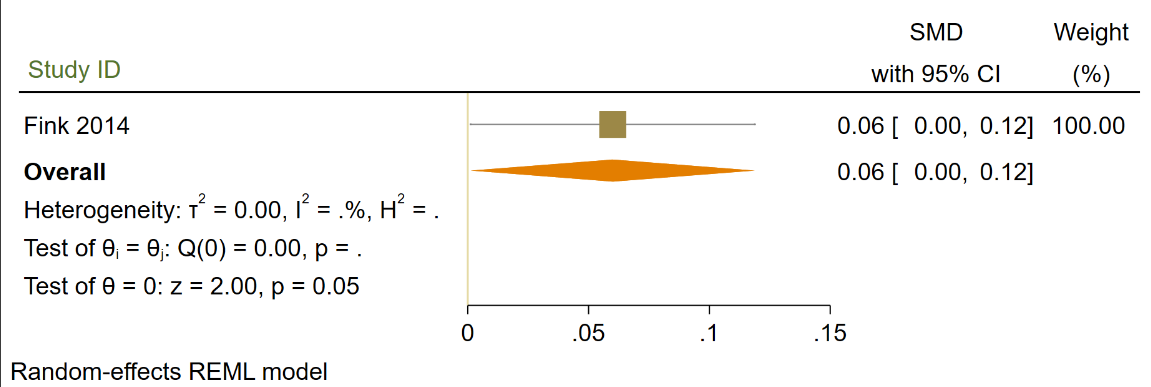
**

**Fig. S9: Baseline age of the height/length for age (HAZ/LAZ) assessment among the included studies**

**
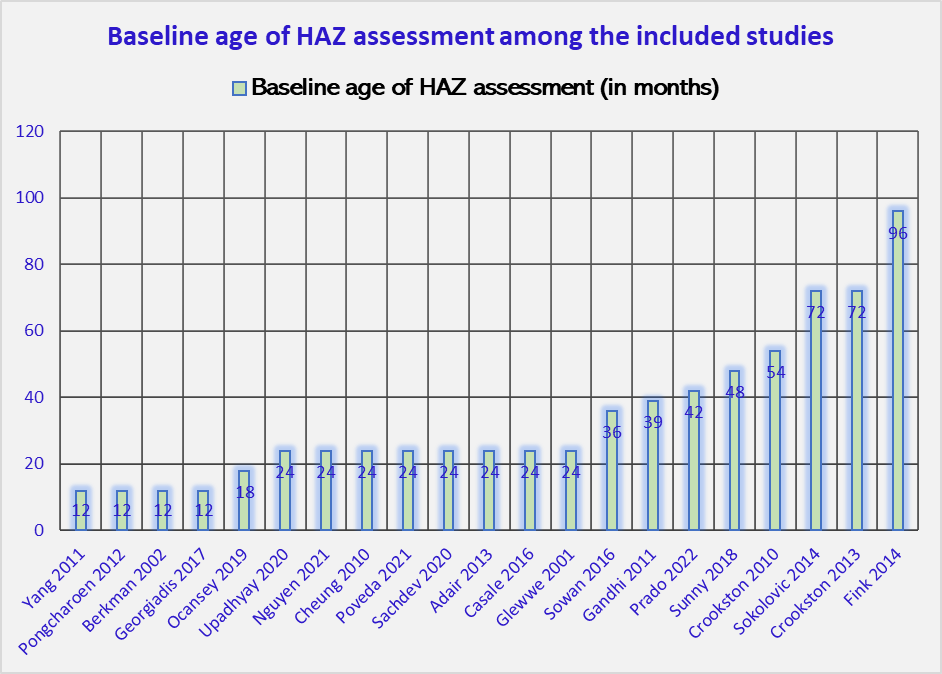
**
